# Supplementary material for: Cross-validation of distance measurements in proteins by PELDOR/DEER and single-molecule FRET
Source: Nat Commun. 2022 Jul 29;13:4396. doi: 10.1038/s41467-022-31945-6 (PMC9338047; doi:10.1038/s41467-022-31945-6)
Supplement: Supplementary file 1 — Supplementary Information [file 41467_2022_31945_MOESM1_ESM.pdf]

Supplementary information for:

Cross-validation of distance measurements in proteins by PELDOR/DEER and single-molecule FRET

Martin F. Peter[1,#], Christian Gebhardt[2,#], Rebecca Mächtel[2], Gabriel G. Moya Muñoz[2], Janin Glaenzer[1], Alessandra Narducci[2], Gavin H. Thomas[3], Thorben Cordes[2,*], Gregor Hagelueken[1,*]

[1]Institute of Structural Biology, University of Bonn, Bonn, Germany

[2]Physical and Synthetic Biology, Faculty of Biology, Ludwig-Maximilians-Universität München, Planegg-Martinsried, Germany

[3]Department of Biology (Area 10), University of York, York, United Kingdom

*Corresponding author emails: cordes@bio.lmu.de, hagelueken@uni-bonn.de

#equal contribution

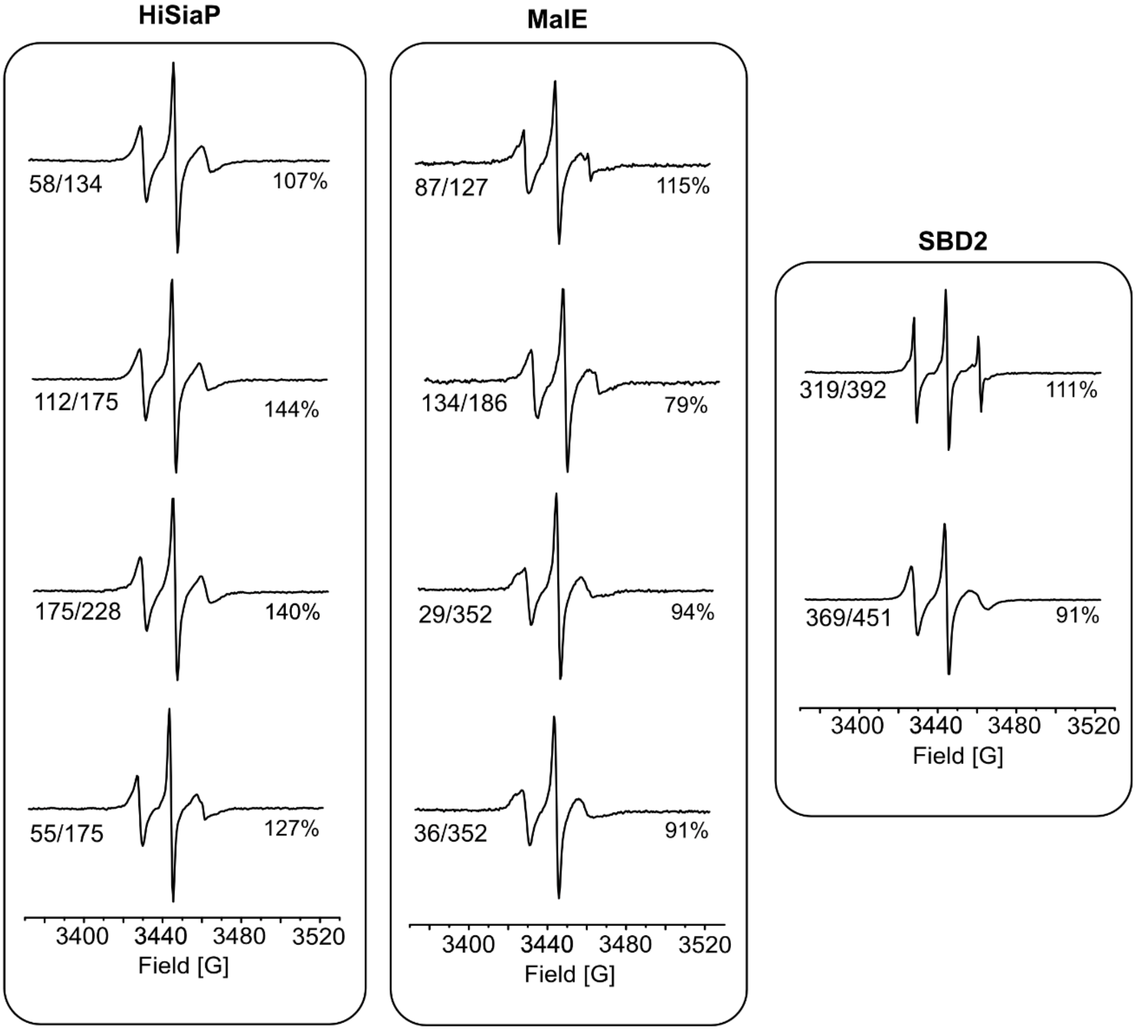

Supplementary Fig. 1: Room temperature cw-EPR spectra of spin labelled double variants. The labelling of each variant with MTSSL was verified with room temperature cw-EPR spectroscopy (X-band). The labelling efficiencies were determined with the spectrometer software and are given next to each spectrum. Source data are provided as a Source Data file.

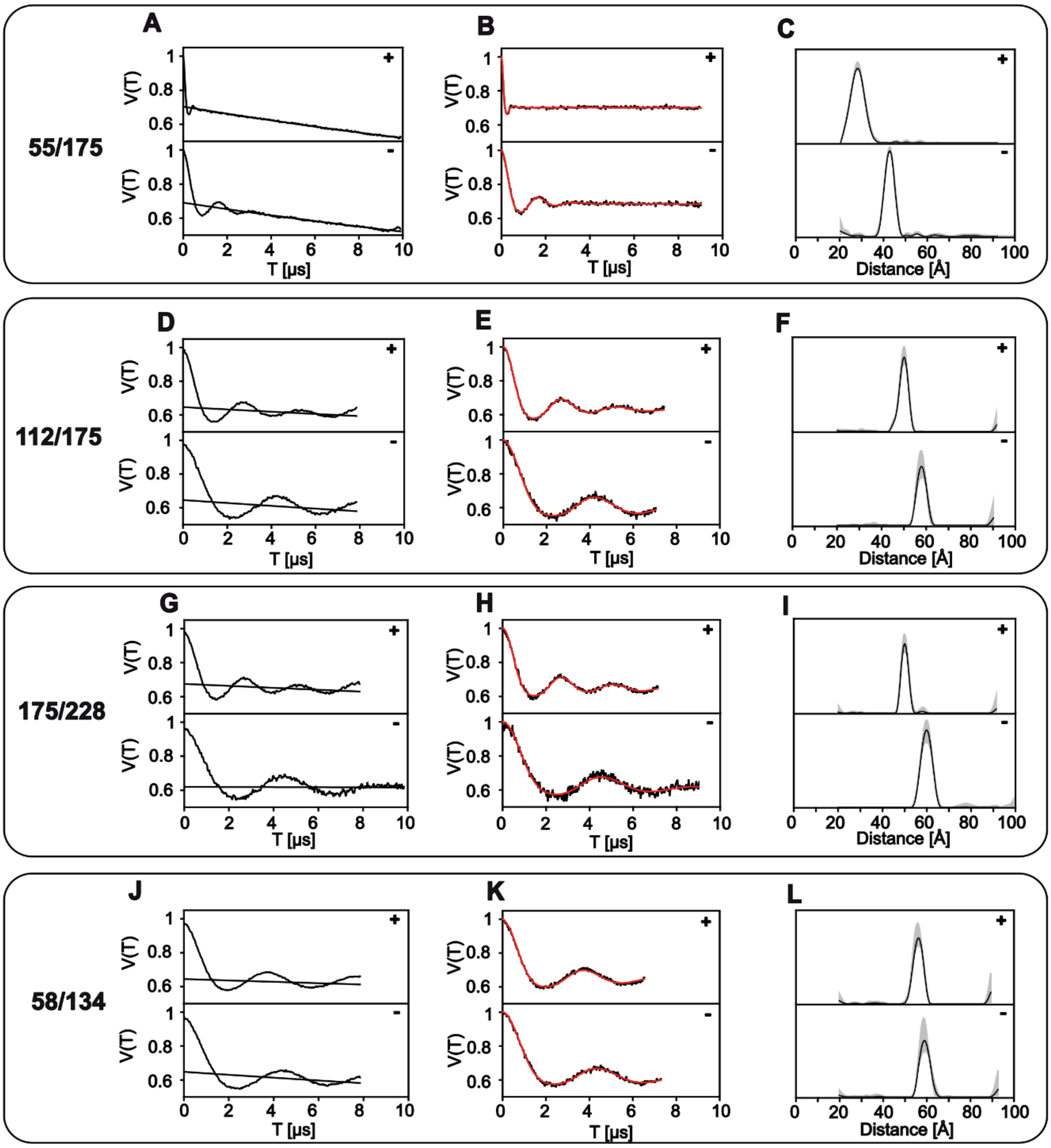

Supplementary Fig. 2: PELDOR/DEER data of HiSiaP variants. A, D, G, J) Raw PELDOR/DEER time traces for apo (-) and holo (+) measurements of each double variant. The background fit is indicated as a black line. **B, E, H, K)** Background-corrected PELDOR/DEER time traces (black) and fits of the signal (red). **C, F, I, L)** Distance distributions from PELDOR/DEER time traces (black). The grey shade around the PELDOR/DEER data is the error margin calculated using the validation tool of DeerAnalysis [1]. Source data are provided as a Source Data file.

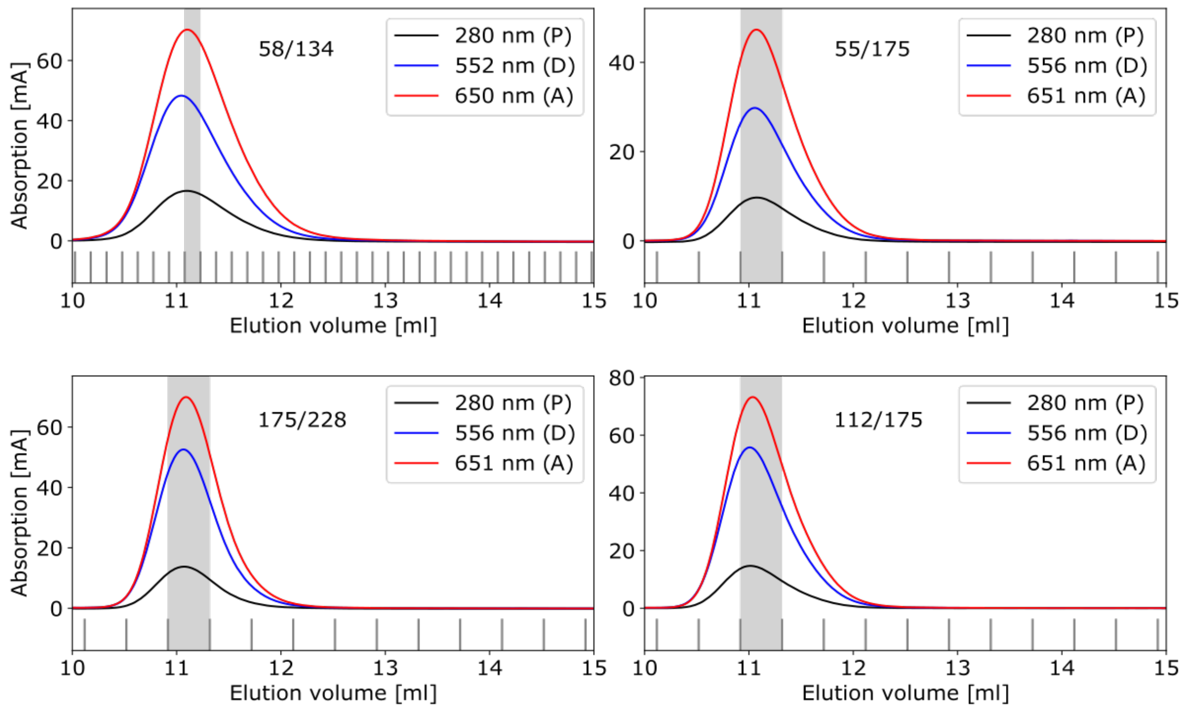

Supplementary Fig. 3: Size exclusion chromatography of HiSiaP variants labelled with Alexa Fluor 555 – Alexa Fluor 647. Absorption profile of the size extrusion chromatography (ÄKTA, Superdex 75 Increase 10/300 GL, GE Healthcare) for all tested HiSiaP variants 58/134, 175/228, 55/175 and 112/175 to monitor protein concentration (280 nm) and Alexa Fluor 555 (552 nm) / Alexa Fluor 647 (650 nm). The grey area indicates the fraction used in the smFRET experiments, where labelling efficiencies of >90% was achieved for all samples. Source data are provided as a Source Data file.

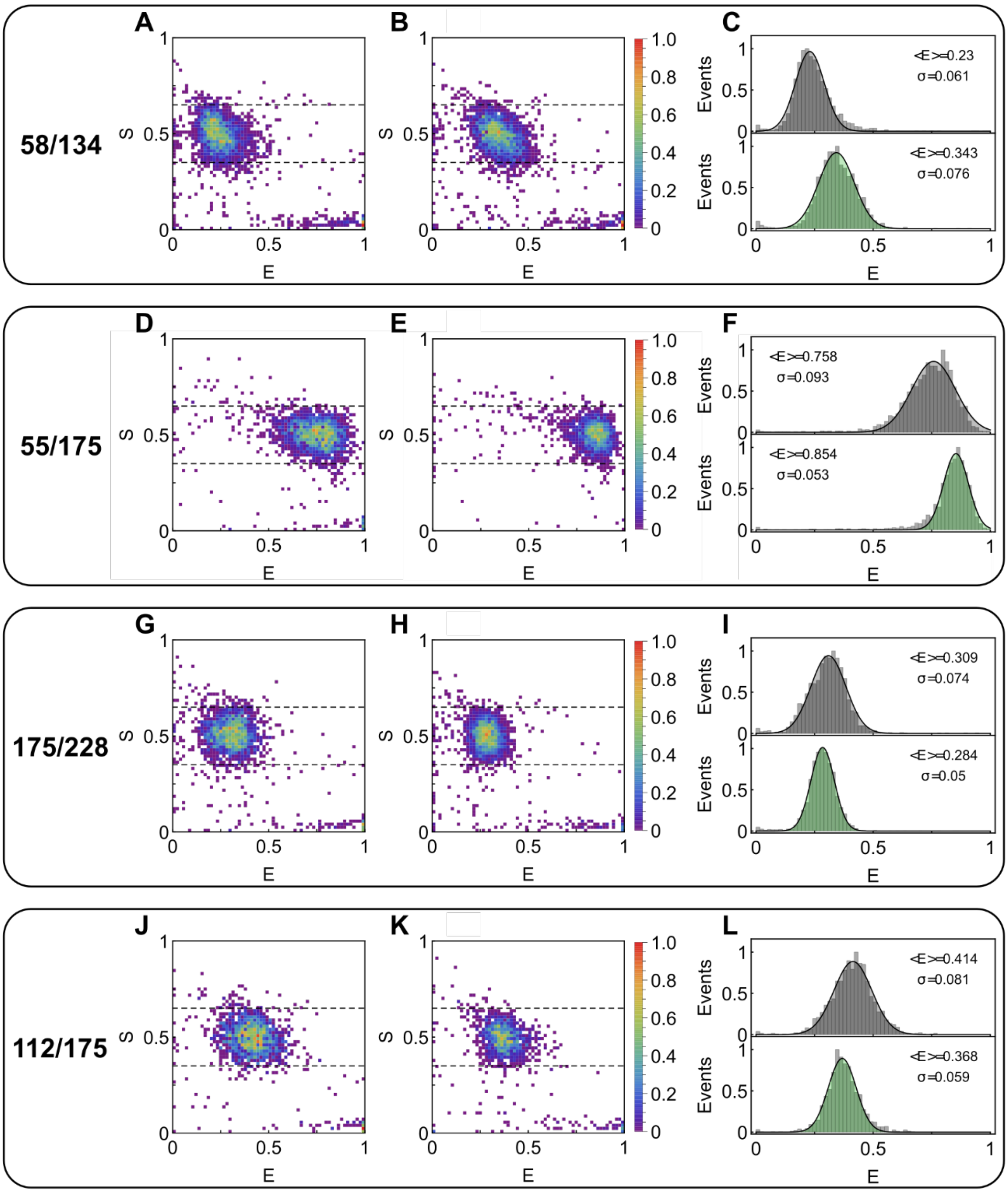

Supplementary Fig. 4: smFRET data of HiSiaP with Alexa Fluor 555 – Alexa Fluor 647. A, C, E, G) ES-2D-Histograms of HiSiaP variants 58/134, 175/228, 30/175, 55/175, and 112/175 in apo state (-) and holo state (+). **B, D, F, H)** 1D-E-Histograms extracted from the ES-data for apo (grey) and holo (green) are fitted with a 1D-Gaussian distribution. Mean <E> and standard deviation σ are labelled. The numbers of considered bursts N in apo/holo states are (C) 2435/2316, (F) 2129/1777, (I) 1932/2082, and (L) 1529/1367, respectively. Source data are provided as a Source Data file.

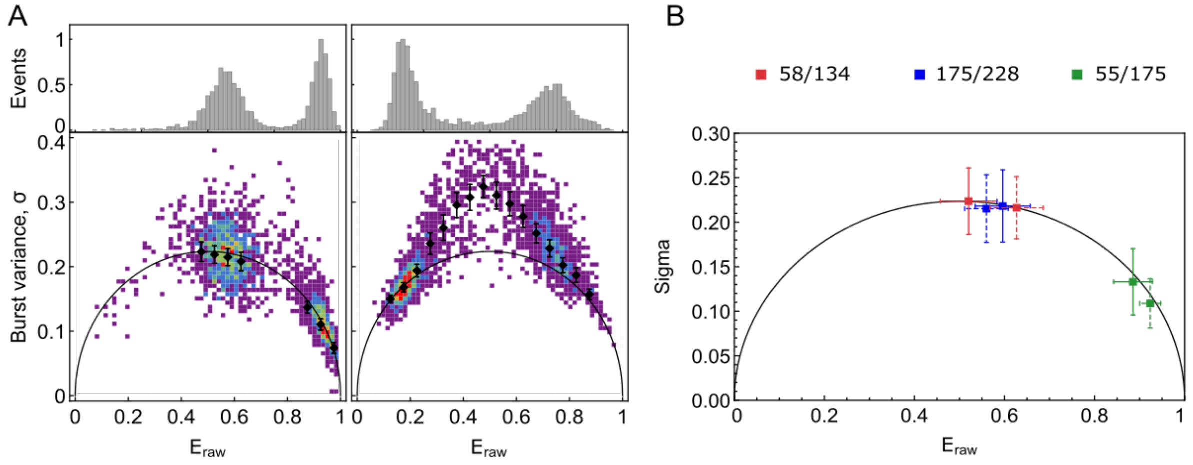

Supplementary Fig. 5: Burst-variance analysis of HiSiaP ALEX data. A) Burst variance analysis example of 55/175 and 175/228 in their holo states (left, joined data set, n=2718/1933) and dynamic control experiments with a fluctuating DNA-hairpin (right, n=2824) from ref. [2]. Data are binned into bins of 0.05 and mean plus/minus SD are shown (black). **B)** Population mean (n=3) and standard deviation of all bursts of one measurement of burst variance analysis for three HiSiaP variants from Figure 3 in apo (solid) and holo state (dashed). Source data are provided as a Source Data file.

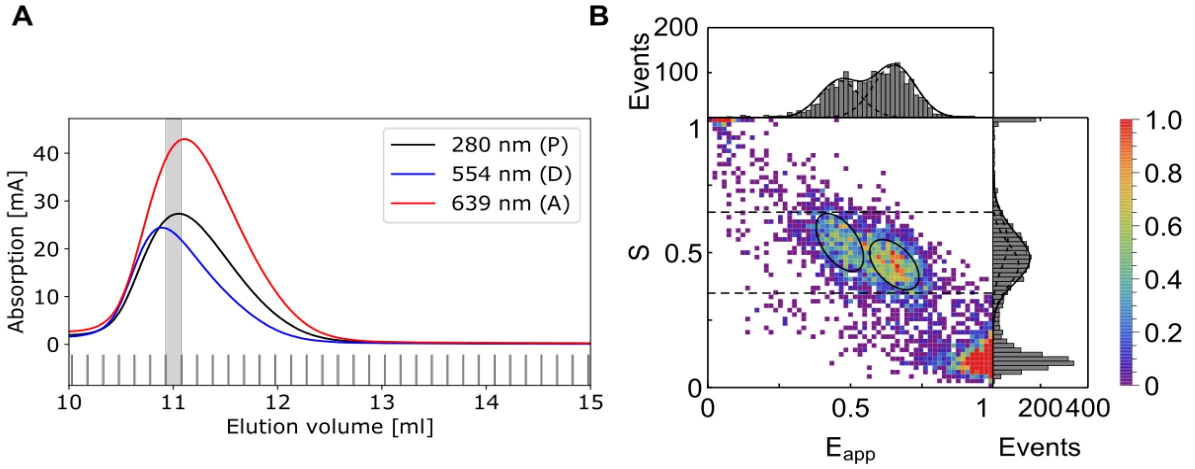

Supplementary Fig. 6: Extreme example for environmental effect on FRET efficiency. HiSiaP variant 112/175 labelled with Alexa Fluor546 – Star635P. **A)** Absorption profile of the size extrusion chromatography (ÄKTA, Superdex 75 Increase 10/300 GL, GE Healthcare) to monitor protein concentration (280 nm) and Alexa Fluor546 (554 nm) / Star635P (639 nm). The grey area indicates the fraction used in the smFRET experiments, where labelling efficiencies of >90%. **B)** ES-2D-Histograms of variant from A) in apo state. 1D-E-Histograms and 1D-S-Histogram are shown on top and on the right, respectively. The ES-data are fitted with a 2D-Gaussian distribution where the 1D-integrals are shown in the 1D-histograms (black lines). Source data are provided as a Source Data file.

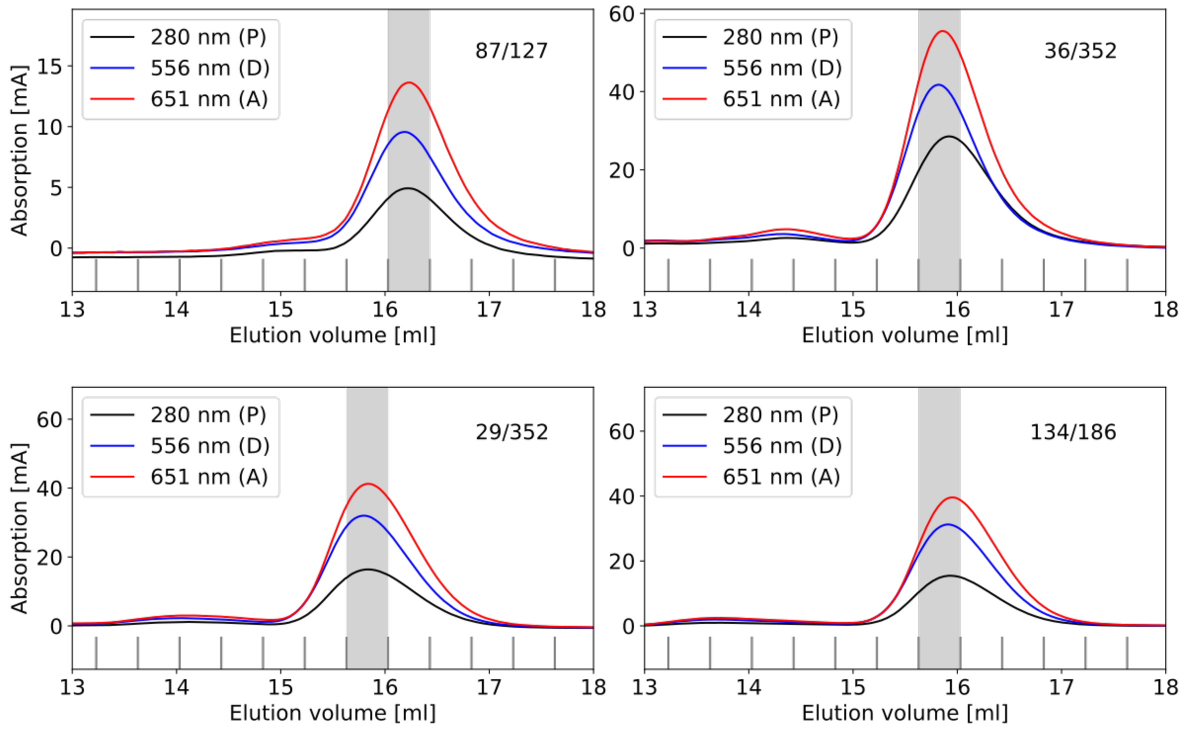

Supplementary Fig. 7: Size exclusion chromatography of MalE variants labelled with Alexa Fluor 555 – Alexa Fluor 647. Absorption profile of the size extrusion chromatography (ÄKTA, Superdex 75 Increase 10/300 GL, GE Healthcare) for all tested MalE variants 87/127, 36/352, 29/352 and 134/186 to monitor protein concentration (280 nm) and Alexa Fluor 555 (552 nm) / Alexa Fluor 647 (650 nm). The grey area indicates the fraction used in the smFRET experiments, where labelling efficiencies of >90% was achieved for all samples. Source data are provided as a Source Data file.

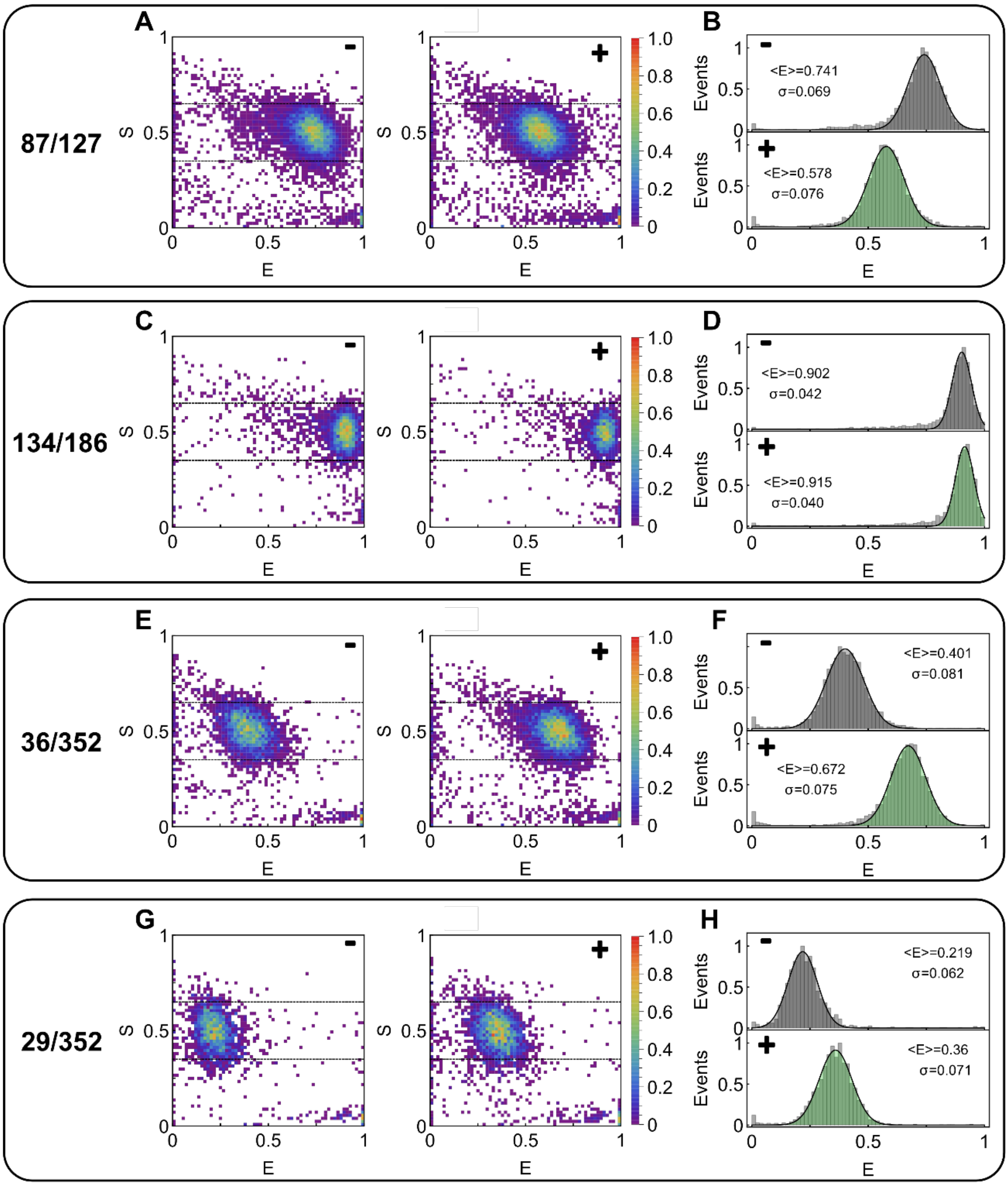

Supplementary Fig. 8: smFRET data of MalE with Alexa Fluor 555 – Alexa Fluor 647. A, C, E, G) ES-2D-Histograms of MalE variants 87/186, 134/186, 36/352, and 29/352 in apo state (-) and holo state (+). **B, D, F, H)** 1D-E-Histograms extracted from the ES-Data for apo (grey) and holo (green) are fitted with a 1D-Gaussian distribution. Mean <E> and standard deviation σ are labelled. The numbers of considered bursts N in apo/holo states are (B) 6168/6181, (D) 6902/5672, (F) 2593/4293, and (H) 1392/2249, respectively. Source data are provided as a Source Data file.

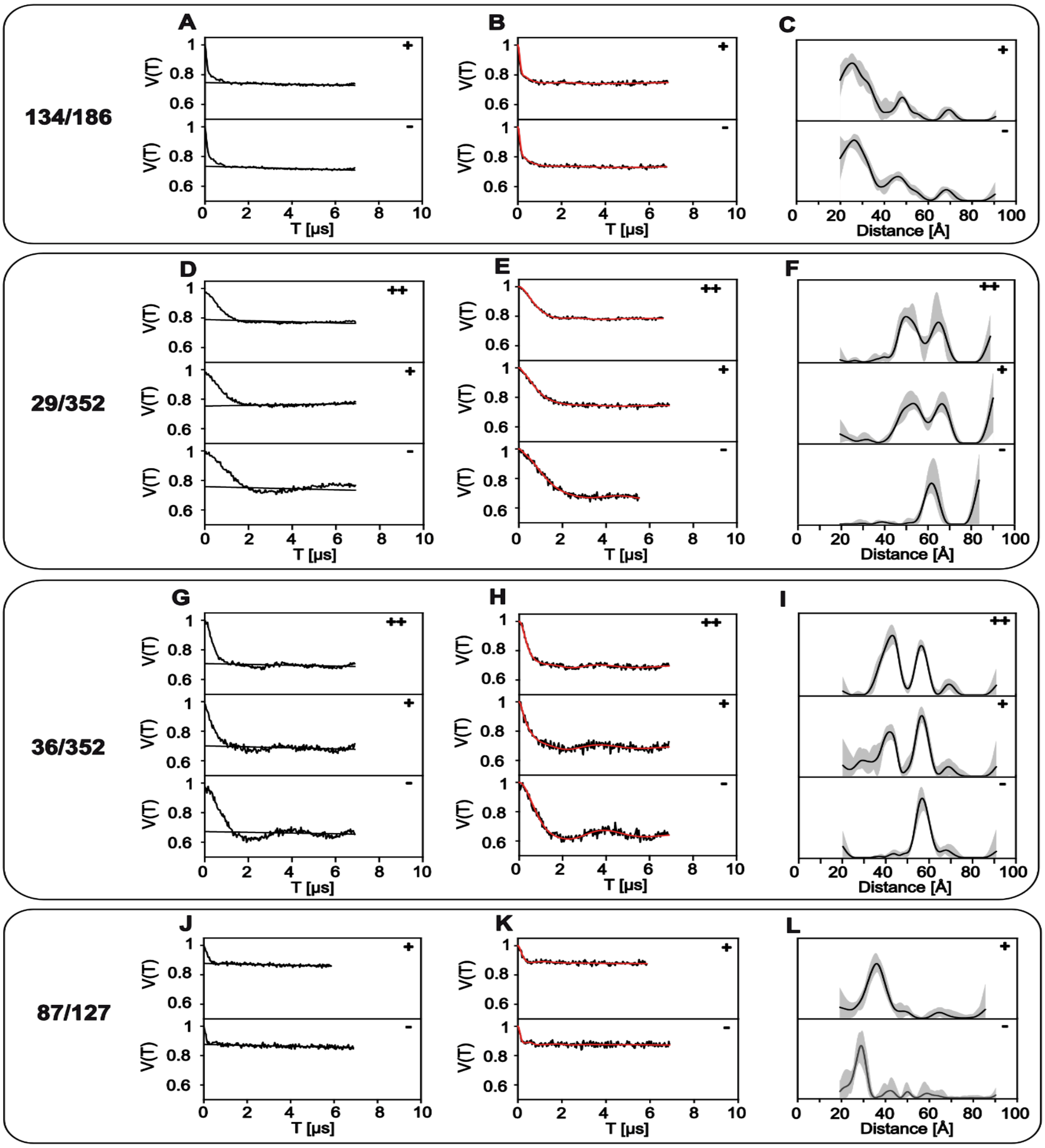

Supplementary Fig. 9: PELDOR/DEER data of MalE variants. A, D, G, J) Raw PELDOR/DEER time traces for apo (-) and holo (+, 1 mM; ++, 10 mM maltose) measurements of each double variant. The background fit is indicated as a black line. **B, E, H, K)** Background-corrected PELDOR/DEER time traces (black) and fits of the signal (red). **C, F, I, L)** Distance distributions from PELDOR/DEER time traces (black).The grey shade around the PELDOR/DEER data is the error margin calculated using the validation tool of DeerAnalysis [1]. Source data are provided as a Source Data file.

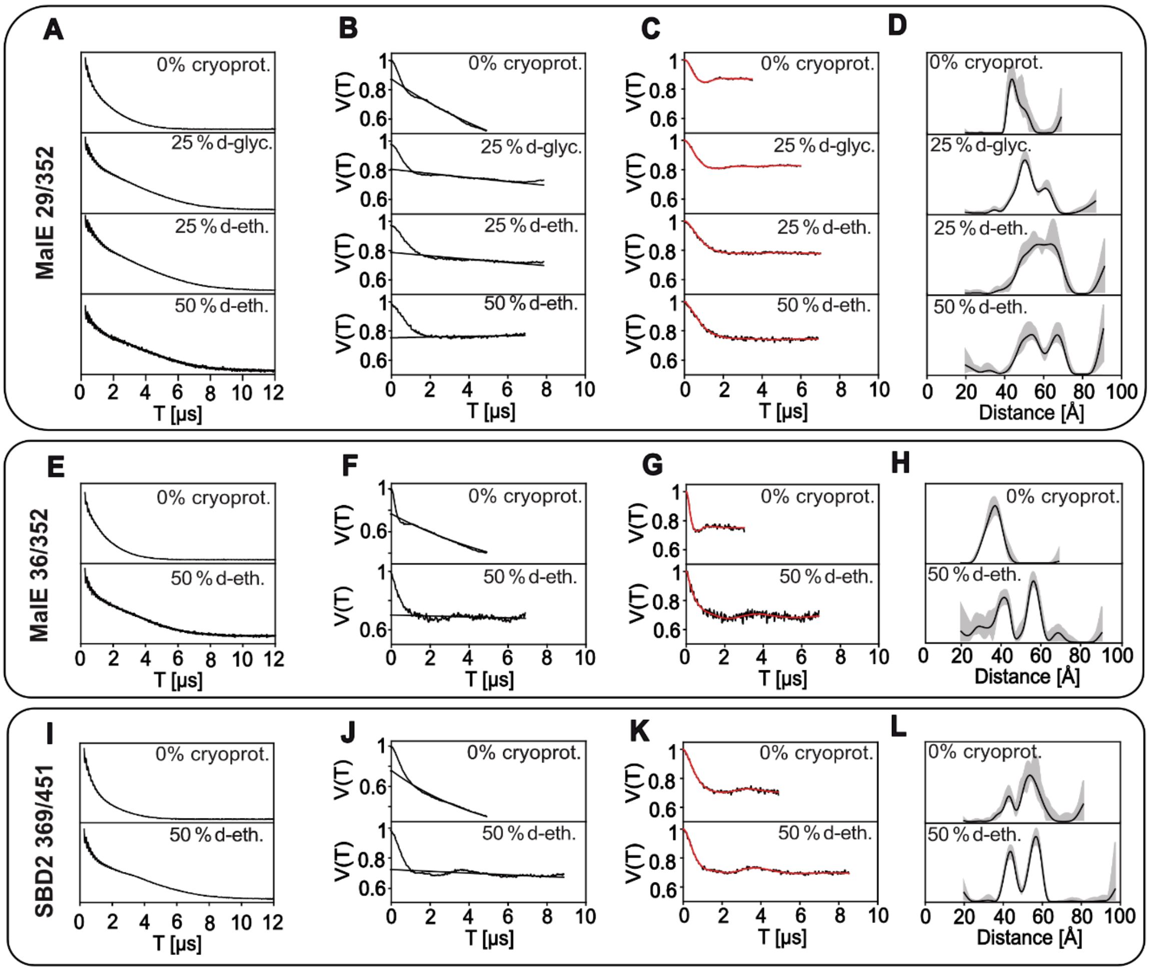

Supplementary Fig. 10: PELDOR/DEER experiments with varying amounts of cryoprotectant. A, E, I) 2PESEEM (2-pulse electron spin echo envelope modulation) spectra for each variant. **B, F, J)** Raw PELDOR/DEER time traces for each measurement. The background fit is indicated as a black line**.** **C, G, K)** Background-corrected PELDOR/DEER time traces (black) and fits of the signal (red). **D, H, L)** Distance distributions from PELDOR/DEER time traces (black). The grey shade around the PELDOR/DEER data is the error margin calculated using the validation tool of DeerAnalysis [1]. Source data are provided as a Source Data file.

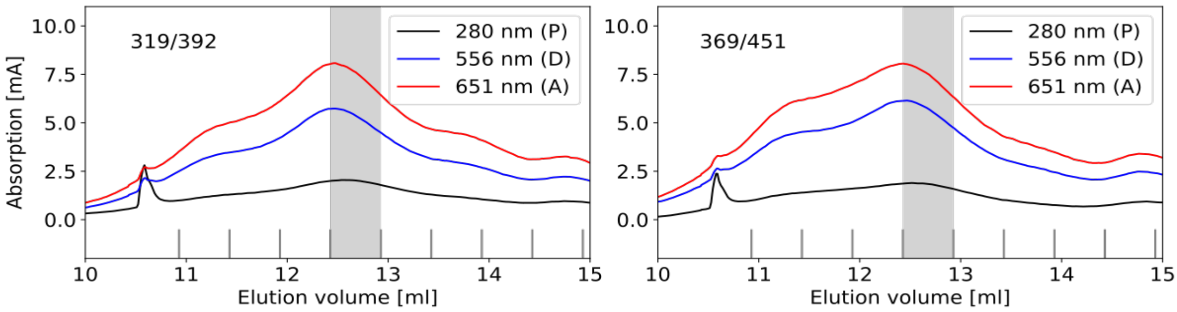

Supplementary Fig. 11: Size exclusion chromatography of SBD2 variants labelled with Alexa Fluor 555 – Alexa Fluor 647. Absorption profile of the size extrusion chromatography (ÄKTA,

Superdex 75 Increase 10/300 GL, GE Healthcare) for all tested SBD2 variants 319/392 and 369/451 to monitor protein concentration (280 nm) and Alexa Fluor 555 (552 nm) / Alexa Fluor 647 (650 nm). The grey area indicates the fraction used in the smFRET experiments, where labelling efficiencies of >90% was achieved for all samples. Source data are provided as a Source Data file.

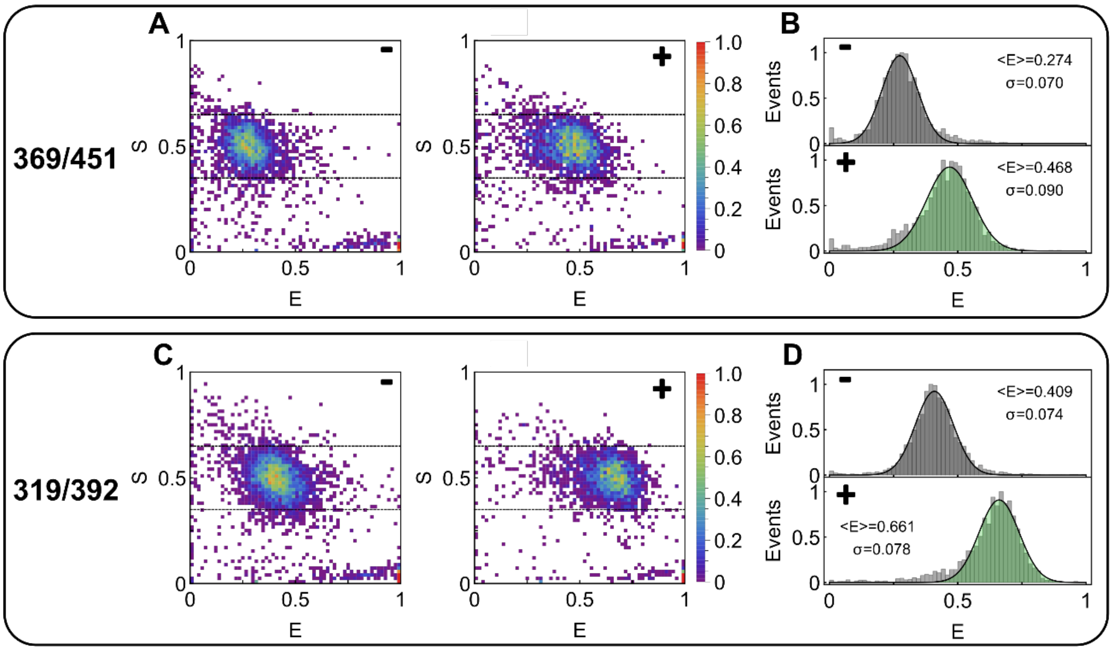

Supplementary Fig. 12: smFRET data of SBD2 with Alexa Fluor 555 – Alexa Fluor 647. A, C) ES-2D-Histograms of SBD2 variants 369/451 and 319/392 in apo state (-) and holo state (+). **B, D)** 1D-E-Histograms extracted from the ES-Data for apo (grey) and holo (green) are fitted with a 1D-Gaussian distribution. Mean <E> and standard deviation σ are labelled. The numbers of considered bursts N in apo/holo states are (B) 2050/2189 and (D) 3377/2060, respectively. Source data are provided as a Source Data file.

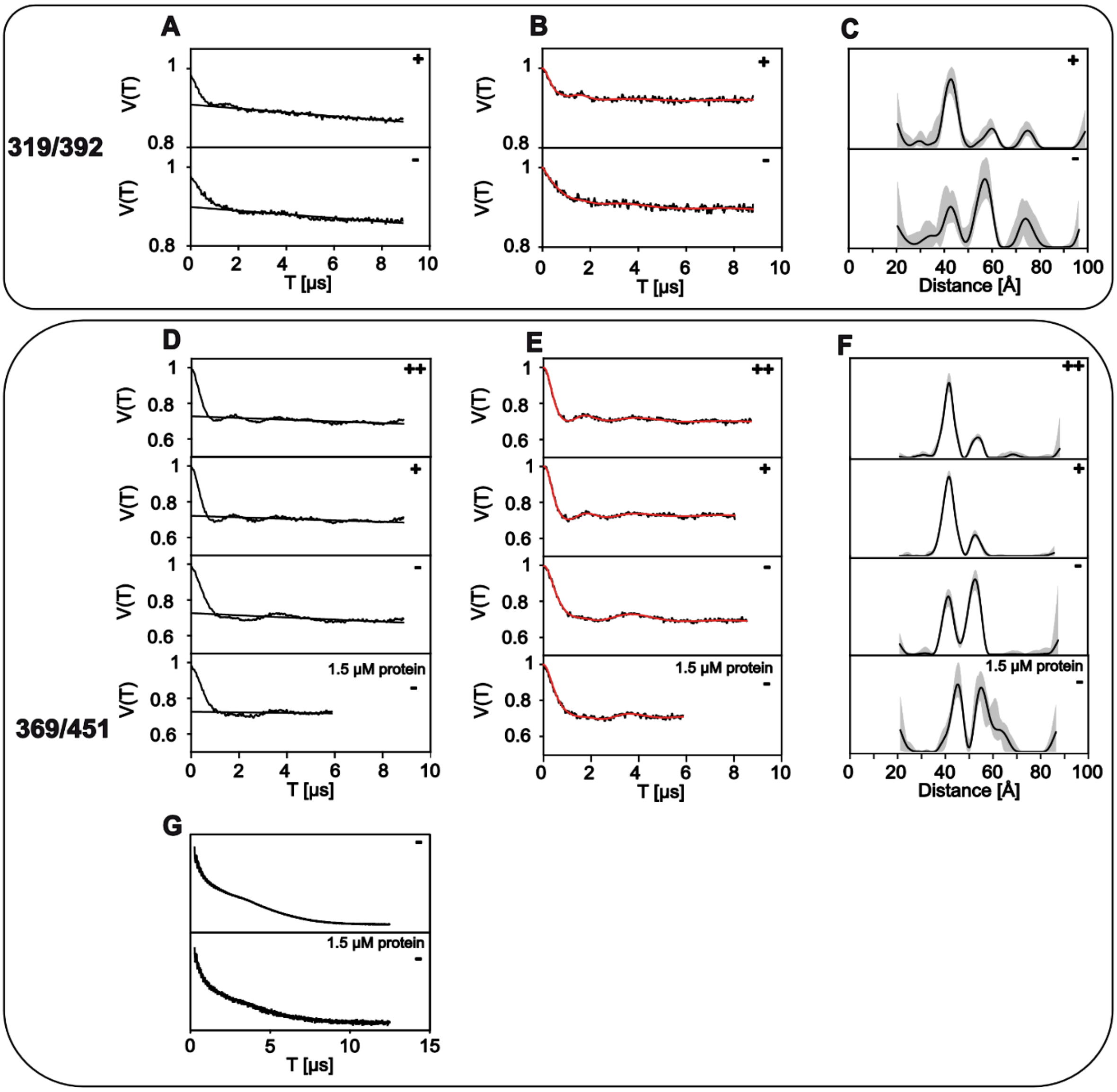

Supplementary Fig. 13: PELDOR/DEER data of SBD2 variants. A, D) Raw PELDOR/DEER time traces for apo (-) and holo (+ and ++) measurements of each double variant. The background fit is indicated as a black line. **B, E)** Background-corrected PELDOR/DEER time traces (black) and fits of the signal (red). **C, F)** Distance distributions from PELDOR/DEER time traces (black). The grey shade around the PELDOR/DEER data is the error margin calculated using the validation tool of DeerAnalysis [1]. **G)** 2PESEEM spectra of apo measurements for 369/451 for 15 µM and 1.5 µM protein concentrations. Source data are provided as a Source Data file.

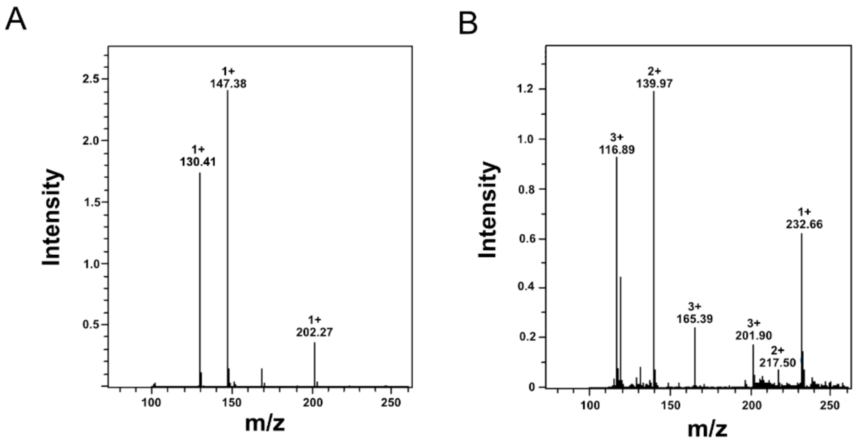

Supplementary Fig. 14: LC-MS for detection of glutamine. A) Mass spectrum of glutamine in standard protein buffer as positive control ($MW_{glutamine}$: 146.15 g/mol). **B)** Mass spectrum of supernatant after precipitation and centrifugation of SBD2 protein sample.

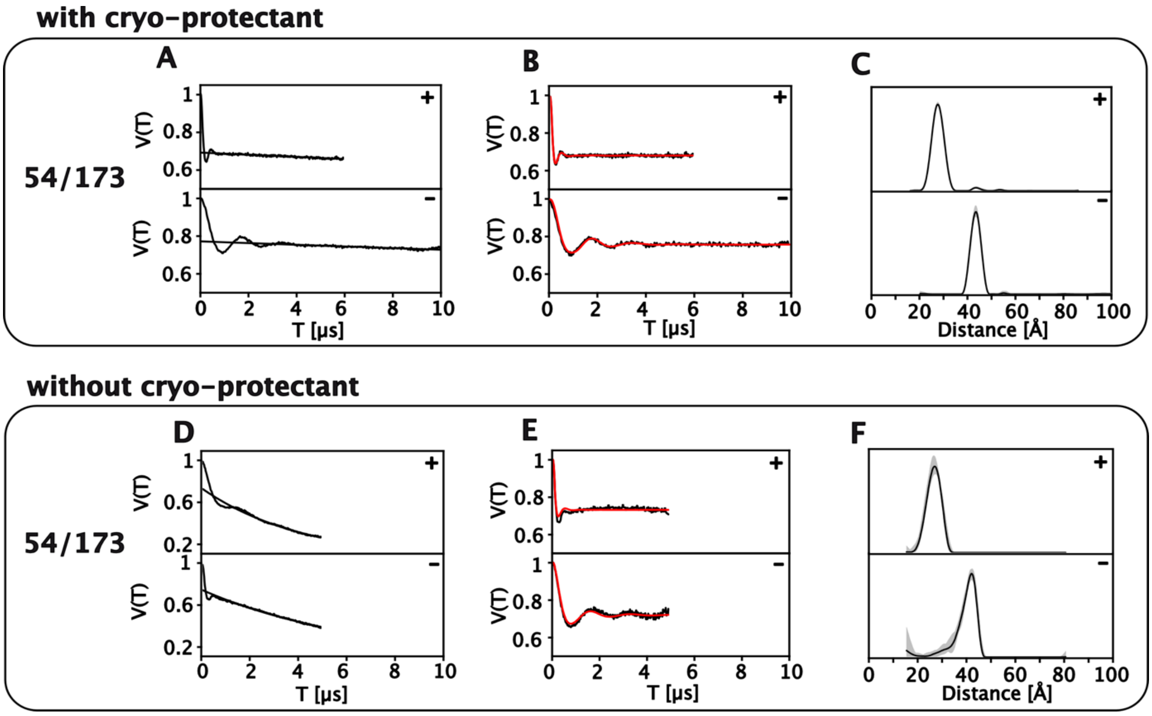

Supplementary Fig. 15: Comparison of PELDOR/DEER measurements on SiaP from *V. cholerae* with and without cryo-protectant. A, D) Raw PELDOR/DEER time traces for apo (-) and holo (+, 1 mM Neu5Ac) measurements of each double variant. The background fit is indicated as a black line. **B,**

E) Background-corrected PELDOR/DEER time traces (black) and fits of the signal (red). **C, F)** Distance distributions from PELDOR/DEER time traces (black). The grey shade around the PELDOR/DEER data is the error margin calculated using the validation tool of DeerAnalysis [1]. Source data are provided as a Source Data file.

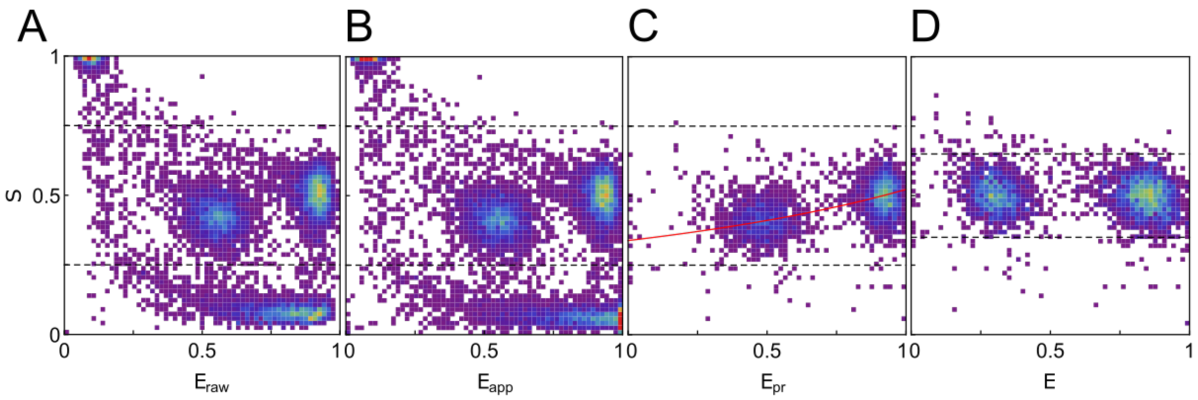

Supplementary Fig. 16: Correction procedure for smFRET to obtain setup-independent FRET efficiencies. A) Raw data in ES-histogram. **B)** Background corrected data. **C)** Removal of donor and acceptor only species and correction for spectral overlap of donor and acceptor fluorophore. The red line indicates the fit of the detection efficiency and quantum efficiency ratio of donor and detector, which leads to slope in S (along E). **D)** Correction for detection and quantum efficiencies (gamma correction); all according to Hellenkamp et al. [3] Source data are provided as a Source Data file.

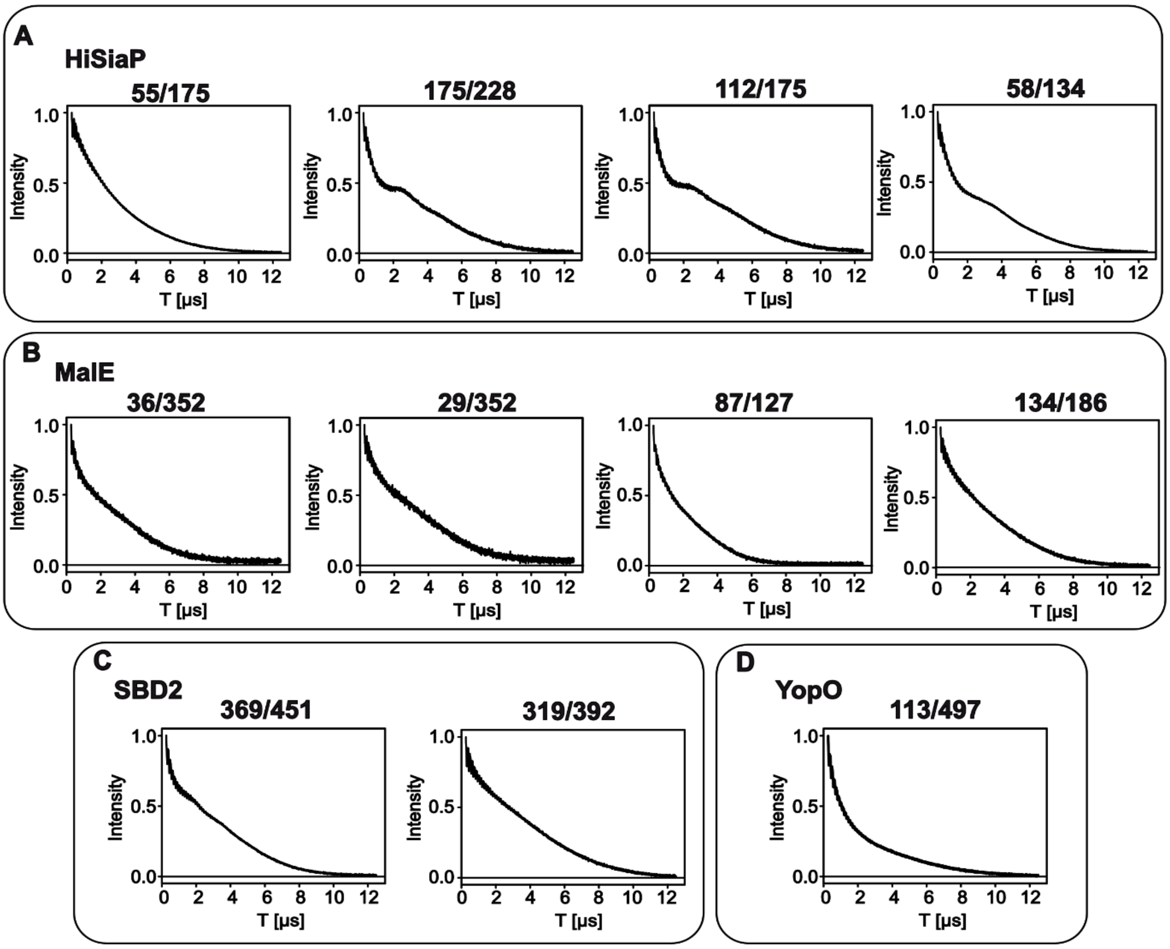

Supplementary Fig. 17: 2pulse-ESEEM spectra for all double spin labelled proteins. A) 2p-ESEEM spectra for spin labelled HiSiaP double variants. **B-D)** Same as A) bur for MalE, SBD2 and YopO spin labelled variants. Source data are provided as a Source Data file.

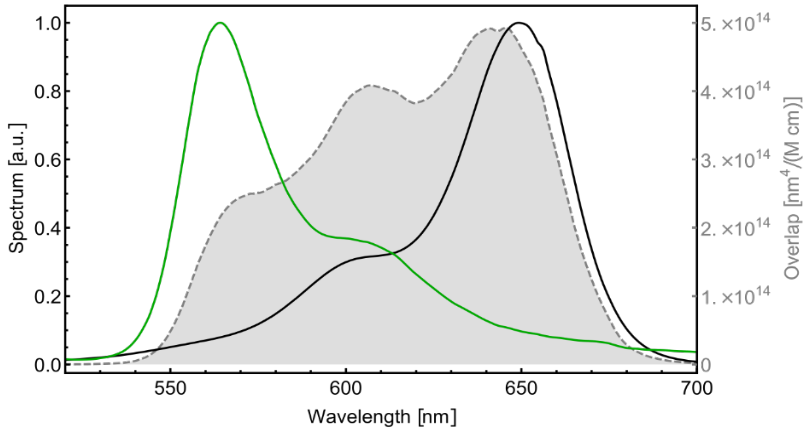

Supplementary Fig. 18: Förster radius determination. Normalized emission spectrum of Alexa Fluor 555 (green) and normalized absorbance spectrum Alexa Fluor 647 (black) determine the spectral overlap integral (grey area). Source data are provided as a Source Data file.

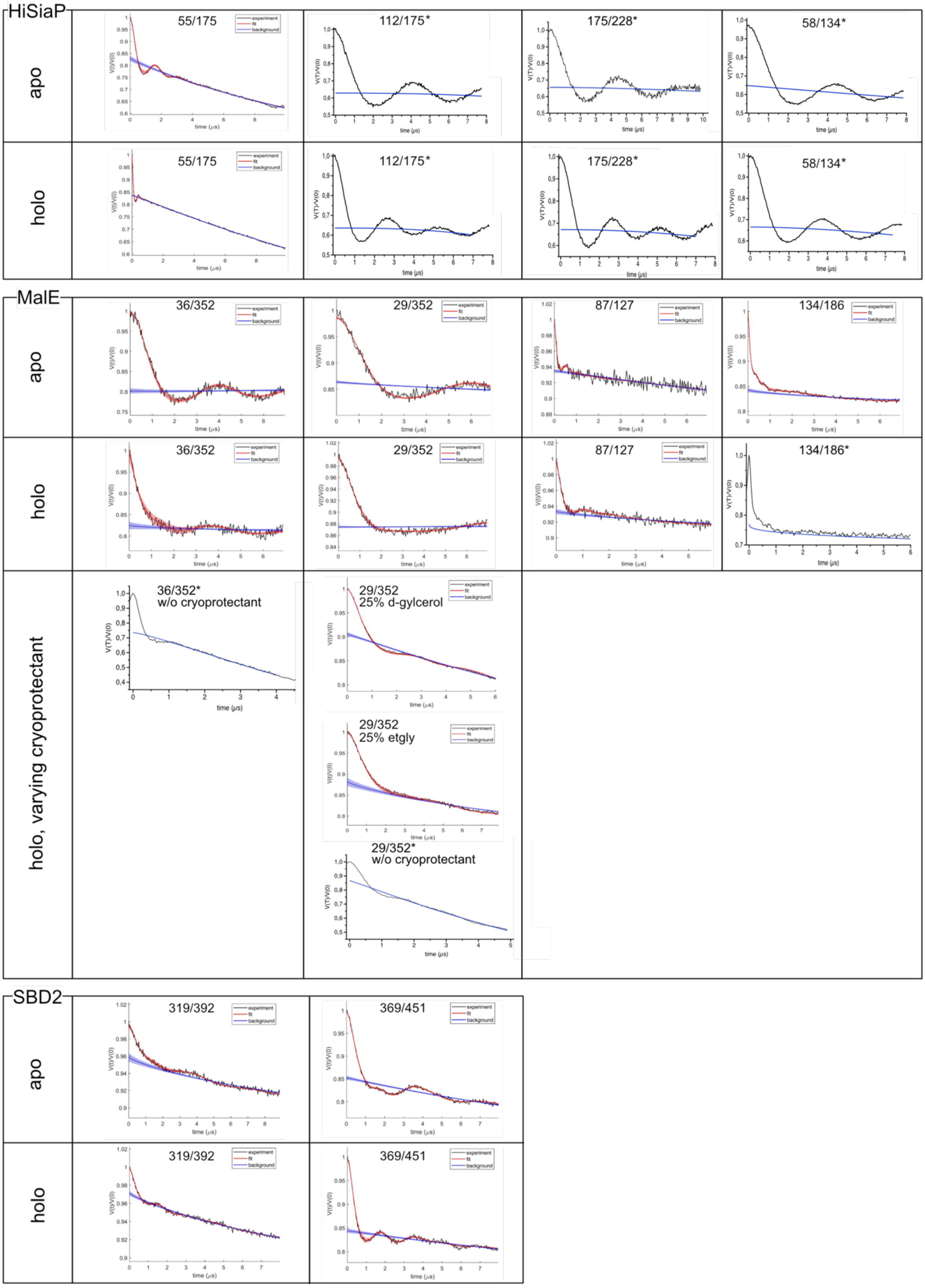

Supplementary Fig. 19: Time traces from comparisons #1-3 processed with DeerNet. The data was processed with the ComparativeDeerAnalyzer on a Windows laptop. In some cases, the software ended with an error message (marked with asterisks). We then used DeerNet as implemented in DeerAnalysis2019, which processed the data without any errors. The blue shade represents the uncertainty of the background fit.

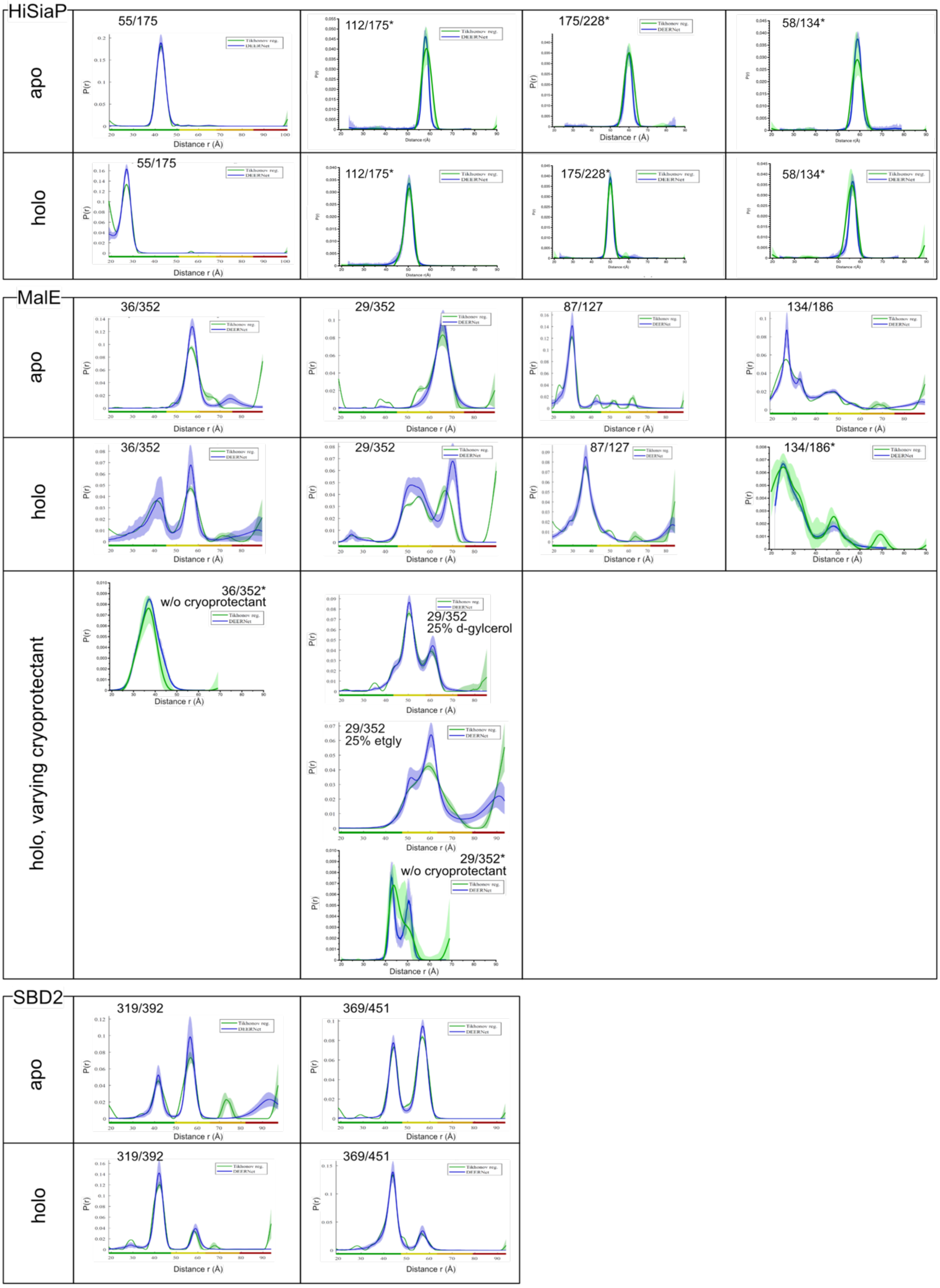

Supplementary Fig. 20: Distance distributions from comparisons #1-3 calculated with DeerNet. The data was processed with the ComparativeDeerAnalyzer on a Windows laptop. The DeerNet result is shown in blue, the result form Thikonov regularization in green. Blue and green shades represent the error margins for the two approaches. In some cases, the software ended with an error message (marked with asterisks). We then used DeerNet as implemented in DeerAnalysis2019, which processed the data without any errors.

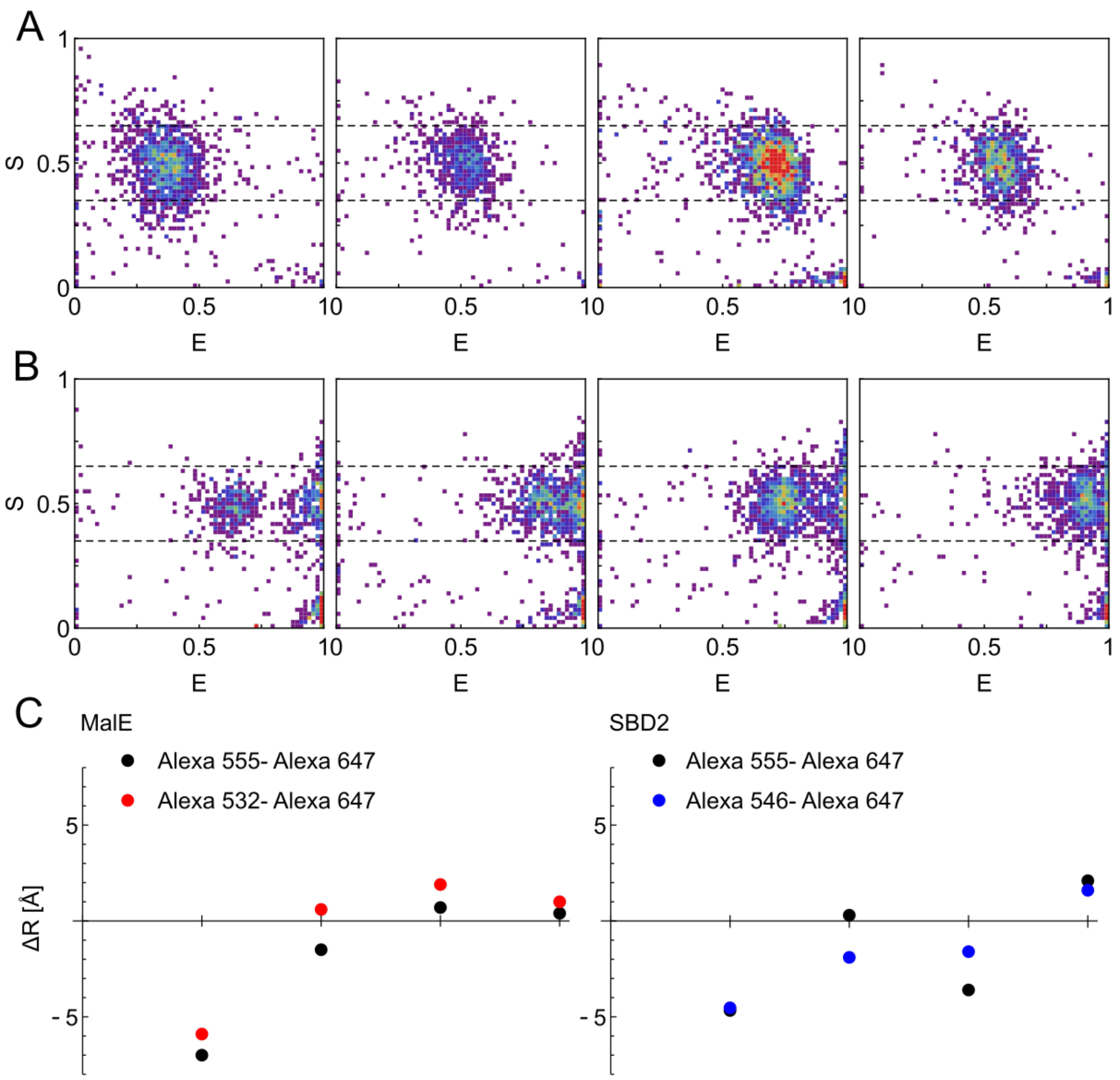

Supplementary Fig. 21: smFRET data of MalE with Alexa Fluor 532 – Alexa Fluor 647 and SBD with Alexa Fluor 546 – Alexa Fluor 647. A) ES-2D-Histograms of MalE variant 29/352 in apo/holo state and variant 87/186 in apo/holo state (left to right). The numbers of considered bursts N are 1532/863/1286/733 (left to right). **B)** ES-2D-Histograms of SBD2 variant 369/451 in apo/holo state and variant 319/392 in apo/holo state (left to right). The numbers of considered bursts N are 559/919/1509/1108 (left to right). **C)** Deviation of calculated distance and simulated distance for MalE (left) and SBD2 (right) for the four measurement conditions show only small variations (<2 Å) for different fluorophores (see Supplementary Table 4). Source data are provided as a Source Data file.

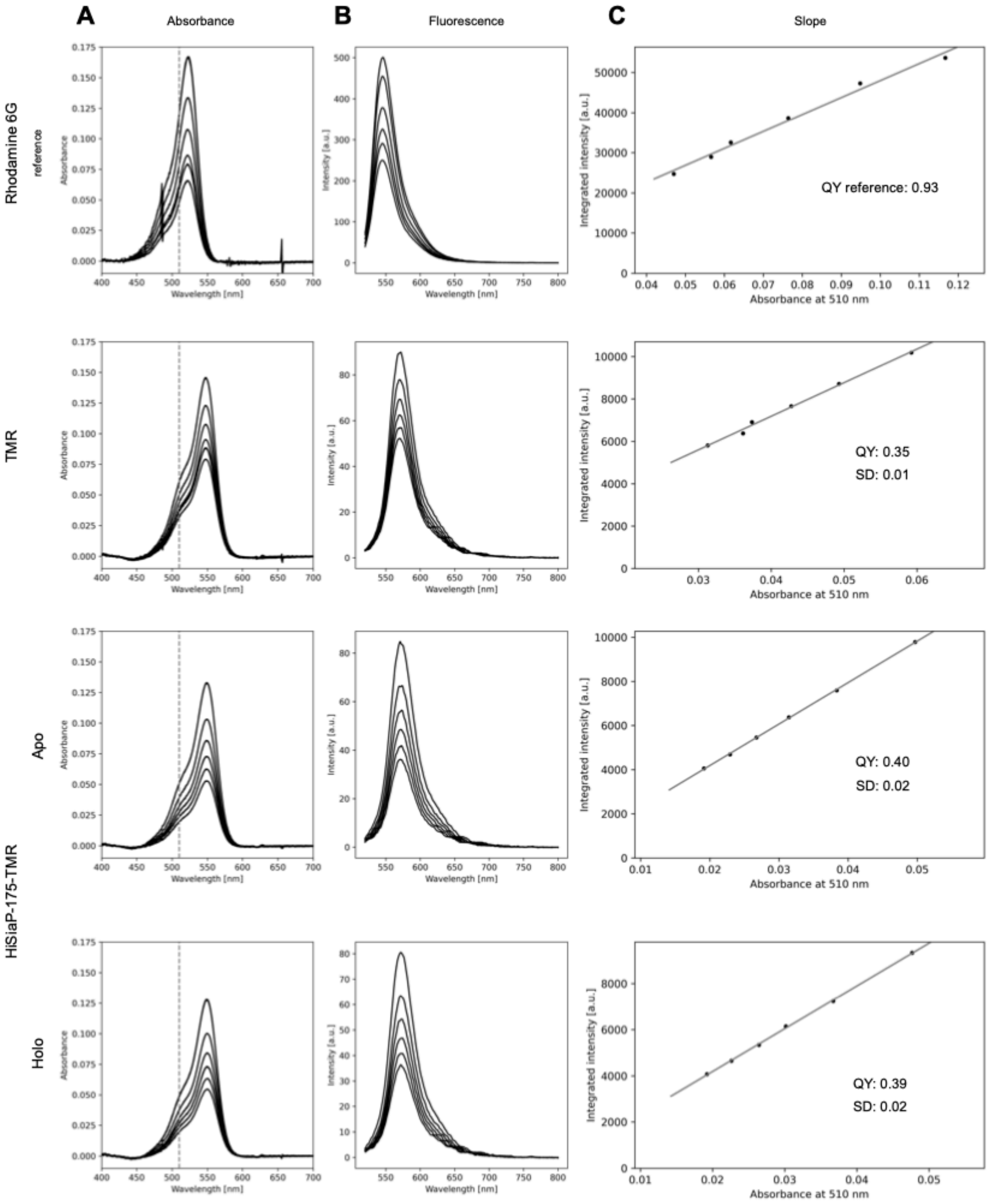

Supplementary Fig. 22: Quantum yield measurement of TMR in reference to Rhodamine 6G. A) Absorbance spectra of Rhodamine 6G (top), TMR (middle), HiSiaP-175-TMR in apo and holo states (bottom) at 6 different concentrations. The dashed line indicates the extracted absorbance values from the excitation wavelength at 510 nm. **B)** Emission spectra of Rhodamine 6G (top), TMR (middle), HiSiaP-175-TMR in apo and holo states (bottom) of the sample in (A) excited at 510 nm. **C)** The integrated emission from (B) is plotted against the absorbance extracted from A and fitted to the function $I_{int} = m*A$. Triplicates were used to determine the quantum yield (QY). Source data are provided as a Source Data file.

Supplementary Table 1: Width and mean positions of smFRET experiments in comparison to the expected width based on pure statistical noise ("shot-noise"). The width (standard deviation) of the FRET histogram is obtained from a Gaussian fit to the uncorrected FRET data. The theoretical width is calculated based on the photon count histograms assuming pure statistical noise.

**Sample**	***Width of E_{raw} data***	***Theoretical width***	***Ratio***
HiSiaP 58/134 apo	0.075	0.04	1.88
HiSiaP 58/134 holo	0.073	0.039	1.87
HiSiaP 55/175 apo	0.047	0.023	2.04
HiSiaP 55/175_holo	0.024	0.02	1.20
HiSiaP 112/175 apo	0.067	0.037	1.81
HiSiaP 112/175_holo	0.054	0.039	1.38
HiSiaP 175/228 apo	0.07	0.039	1.79
HiSiaP 175/228 holo	0.05	0.039	1.28
MalE 87/127 apo	0.047	0.034	1.38
MalE 87/127 holo	0.059	0.041	1.44
MalE 36/352 apo	0.075	0.048	1.56
MalE 36/352_holo	0.054	0.038	1.42
MalE 29/352 apo	0.072	0.048	1.50
MalE 29/352 holo	0.07	0.048	1.46
MalE 134/186 apo	0.033	0.025	1.32
MalE 134/186_holo	0.032	0.025	1.28
SBD2 319/392 apo	0.075	0.046	1.63
SBD2 319/392_holo	0.07	0.043	1.63
SBD2 369/451 apo	0.066	0.044	1.50
SBD2 369/451 holo	0.049	0.034	1.44
YopO 113/497 apo	0.106	0.046	2.30
YopO_113/497 _holo	0.067	0.046	1.46

Supplementary Table 2: Details for the measurements plotted in Figure 8CDE.

Exp.	Sample	*FRET Exp (Å)*	*FRET sim (Å)*	*PELDOR exp (Å)*	*PELDOR sim (Å)*	*PELDOR exp vs. PELDOR sim (Å)*	*FRET exp vs. FRET sim (Å)*	*FRET exp vs. PELDOR exp (Å)*	*FRET sim vs. PELDOR sim. (Å)*
1	HiSiaP 58/134 apo	64.3	65.6	59.0	62.5	-3.5	-1.3	5.3	3.10
2	HiSiaP 58/134 holo	58.5	59.8	56.0	55	1.0	-1.3	2.5	4.80
3	HiSiaP 55/175 apo	41.5	45.2	42.6	38.1	4.5	-3.7	-1.1	7.10
4	HiSiaP 55/175 holo	38.1	35.8	27.8	24.3	3.5	2.3	10.3	11.50
5	HiSiaP 112/175 apo	52.8	59.3	58.4	56.5	1.9	-6.5	-5.6	2.80
6	HiSiaP 112/175 holo	54.4	52.1	50.4	46.5	3.9	2.3	4.0	5.60
7	HiSiaP 175/228 apo	56.8	65.7	59.5	62.3	-2.8	-8.9	-2.7	3.40
8	HiSiaP 175/228 holo	57.9	57.8	50.1	52.4	-2.3	0.1	7.8	5.40
9	MalE 134/186 apo	40.7	37.9	25.2	28.4	-3.2	2.8	15.5	9.50
10	MalE 29/352 apo	63.8	71.1	62.3	67.8	-5.6	-7.3	1.5	3.30
11	MalE 36/352 apo	54.4	58.8	56.5	57.5	-1.0	-4.4	-2.1	1.30
12	MalE 87/127 apo	42.4	41.5	29.4	31.4	-2.0	0.9	13	10.10
13	MalE 87/127 holo	48.2	48.3	36.4	39.1	-2.7	-0.1	11.8	9.20
14	SBD2 319/392_holo	45.2	44.0	42.3	38.9	3.7	1.2	2.9	5.40
15	SBD2 369/451 holo	51.8	52.1	44.0	47.6	-3.6	-0.3	7.8	4.50

Supplementary Table 3: Correction parameter of FRET measurements with Alexa Fluor 555 – Alexa Fluor 647. Overview of all correction factors for smFRET measurements on HiSiaP, MalE, and YopO.

**System**	***BG_{DD} [kHz]***	***BG_{DA} [kHz]***	***BG_{AA} [kHz]***	***α***	***δ***	***γ***	***β***
HiSiaP	1.33	0.83	0.77	0.084	0.065	2.17	0.82
MalE	1.51	0.99	1.79	0.064	0.059	1.56	1.00
SBD2	1.04	0.76	1.17	0.079	0.072	1.97	0.77
YopO	1.67	0.85	0.93	0.087	0.068	2.26	0.64

Supplementary Table 4: Fluorophore comparison. Overview of simulated and measured distances with varying donor fluorophores for selected mutants

	Simulated distance	Measured distance	Simulated distance	Measured distance
**Mutant**	***Alexa Fluor 555 – Alexa Fluor 647***		***Alexa Fluor 532 – Alexa Fluor 647****	
MalE 29/352, apo	71.1	63.8	72.0	66.1
MalE 29/352, holo	57.7	56.8	59.1	59.7
MalE 87/186, apo	47.5	48.2	50.2	52.1
MalE 87/186, holo	54.2	54.6	57.2	58.2
**Mutant**	***Alexa Fluor 555 – Alexa Fluor 647***		***Alexa Fluor 546 – Alexa Fluor 647*****	
SBD2 369/451, apo	65.1	60.1	65.4	60.8
SBD2 369/451, holo	52.1	51.8	51.2	49.3
SBD2 319/392, apo	58.3	53.9	57.6	56
SBD2 319/392, holo	44.0	45.2	44.2	45.8

*Förster radius: $R_0 = 61$ Å

** Förster radius: $R_0 = 66$ Å

Supplementary Table 5: Förster radius calculation for Alexa Fluor 555 – Alexa Fluor 647 and TMR-Cy5. Overview of all used parameters (measured/literature).

**Pair**	***Alexa Fluor 555 – Alexa Fluor 647***	***TMR – Cy5***
Orientation factor κ^2	2/3	2/3
Average refractive index	1.4	1.4
Extinction coefficient	265,000 1/(M cm)	250,000 1/(M cm)
Quantum yield donor	0.14	0.395*
Overlap integral	$8.12 \times 10^{15}\ nm^4/(M\ cm)$	$1.13 \times 10^{16}\ nm^4/(M\ cm)$
**R_0**	**51 Å**	**62.7 Å**

*Determined experimentally, Supplementary Fig. 22

Supplementary Table 6: Experimental distances of three independent smFRET measurements.

Sample	*FRET Exp 1 (year) [Å]*	*FRET Exp 2 (year) [Å]*	*FRET Exp 3 (year) [Å]*	*FRET Exp mean [Å]*	*Std. Dev. [Å]*
HiSiaP 58/134 apo	62.4 (2019)	64.6 (2021)	65.9 (2019)	64.3	1.8
HiSiaP 58/134 holo	57.8 (2019)	58.2 (2021)	59.4 (2019)	58.5	0.8
HiSiaP 55/175 apo	42.5 (2019)	40.3 (2021)	41.6 (2019)	41.5	1.1
HiSiaP 55/175 holo	38.9 (2019)	36.9 (2021)	38.4 (2019)	38.1	1.0
HiSiaP 112/175 apo	55.1 (2019)	51.3 (2021)	52.1 (2022)	52.8	2.0
HiSiaP 112/175 holo	56.9 (2019)	53.3 (2021)	53.1 (2022)	54.4	2.1
HiSiaP 175/228 apo	59.6 (2019)	55.3 (2021)	55.4 (2022)	56.8	2.4
HiSiaP 175/228 holo	60.6 (2019)	56.9 (2021)	56.3 (2022)	57.9	2.4
MalE 134/186 apo	39.5 (2018)	40.9 (2022)	41.7 (2018)	40.7	1.1
MalE 134/186 holo	40.1 (2018)	40.9 (2022)	41.9 (2018)	41.0	0.9
MalE 29/352 apo	63.7 (2018)	62.7 (2018)	65.1 (2018)	63.8	1.2
MalE 29/352 holo	56.6 (2018)	55.8 (2018)	57.9 (2018)	56.8	1.1
MalE 36/352 apo	55.2 (2018)	54.6 (2022)	53.5 (2022)	54.4	0.9
MalE 36/352 holo	45.6 (2018)	44.8 (2022)	44.1 (2022)	44.8	0.8
MalE 87/127 apo	43.2 (2018)	41.6 (2022)	42.4 (2022)	42.4	0.8
MalE 87/127 holo	48.9 (2018)	47.4 (2022)	48.4 (2022)	48.2	0.8
SBD2 319/392 apo	54.7 (2018)	54.3 (2018)	52.8 (2022)	53.9	1.0
SBD2 319/392 holo	46.1 (2018)	45.0 (2018)	44.4 (2022)	45.2	0.8
SBD2 369/451 apo	60.5 (2018)	60.3 (2018)	59.5 (2022)	60.1	0.5
SBD2 369/451 holo	52.5 (2018)	51.8 (2018)	51.0 (2022)	51.8	0.7
YopO 113/497 apo	58.3 (2021)	57.1 (2021)	56.7 (2022)	57.3	0.8
YopO 113/497 holo	69.0 (2021)	68.7 (2021)	67.2 (2022)	68.3	1.0

Supplementary Table 7: Geometric Parameters for in silico predictions of FRET labels.

Label	Linker length [Å]	W [Å]	R1 [Å]	R2 [Å]	R3 [Å]
**Alexa Fluor 555 – C2 Maleimide** [4]	21	4.5	8.8	4.2	1.5
**Alexa Fluor 647 – C2 Maleimide** [5]	21	4.5	11	4.7	1.5
**TMR** [5]	12	4.5	6	4.2	1.5
**Cy5** [5]	21	4.5	11	3	1.5

References

1. Jeschke, G., Chechik, V., Ionita, P. & Godt, A. DeerAnalysis2006—a comprehensive software package for analyzing pulsed ELDOR data. *Applied Magnetic Resonance* **30**, 473-498 (2006).
2. Tsukanov, R., Tomov, T. E., Berger, Y., Liber, M. & Nir, E. Conformational dynamics of DNA hairpins at millisecond resolution obtained from analysis of single-molecule FRET histograms. *The Journal of Physical Chemistry B* **117**, 16105-16109 (2013).
3. Hellenkamp, B. et al. Precision and accuracy of single-molecule FRET measurements-a multi-laboratory benchmark study. *Nature Methods* **15**, 669-676 (2018).
4. Gebhardt, C., Lehmann, M., Reif, M., Zacharias, M. & Cordes, T. Molecular and spectroscopic characterization of green and red cyanine fluorophores from the Alexa Fluor and AF series. *bioRxiv* (2020).
5. Kalinin, S. et al. A toolkit and benchmark study for FRET-restrained high-precision structural modeling. *Nature methods* **9**, 1218-1225 (2012).
