# Supplementary material for: Cross-validation of distance measurements in proteins by PELDOR/DEER and single-molecule FRET
Source: Nat Commun. 2022 Jul 29;13:4396. doi: 10.1038/s41467-022-31945-6 (PMC9338047; doi:10.1038/s41467-022-31945-6)
Supplement: Supplementary file 2 — Reporting Summary [file 41467_2022_31945_MOESM2_ESM.pdf]

nature portfolio

Corresponding author(s): Gregor Hagelueken, Thorben Cordes

Last updated by author(s): Jun 23, 2022

Reporting Summary

Nature Portfolio wishes to improve the reproducibility of the work that we publish. This form provides structure for consistency and transparency in reporting. For further information on Nature Portfolio policies, see our Editorial Policies and the Editorial Policy Checklist.

Statistics

For all statistical analyses, confirm that the following items are present in the figure legend, table legend, main text, or Methods section.

n/a	Confirmed	
☐	☒	The exact sample size (*n*) for each experimental group/condition, given as a discrete number and unit of measurement
☐	☒	A statement on whether measurements were taken from distinct samples or whether the same sample was measured repeatedly
☒	☐	The statistical test(s) used AND whether they are one- or two-sided<br>*Only common tests should be described solely by name; describe more complex techniques in the Methods section.*
☒	☐	A description of all covariates tested
☒	☐	A description of any assumptions or corrections, such as tests of normality and adjustment for multiple comparisons
☐	☒	A full description of the statistical parameters including central tendency (e.g. means) or other basic estimates (e.g. regression coefficient) AND variation (e.g. standard deviation) or associated estimates of uncertainty (e.g. confidence intervals)
☒	☐	For null hypothesis testing, the test statistic (e.g. *F*, *t*, *r*) with confidence intervals, effect sizes, degrees of freedom and *P* value noted<br>*Give P values as exact values whenever suitable.*
☒	☐	For Bayesian analysis, information on the choice of priors and Markov chain Monte Carlo settings
☒	☐	For hierarchical and complex designs, identification of the appropriate level for tests and full reporting of outcomes
☒	☐	Estimates of effect sizes (e.g. Cohen's *d*, Pearson's *r*), indicating how they were calculated

Our web collection on statistics for biologists contains articles on many of the points above.

Software and code

Policy information about availability of computer code

Data collection	Bruker Xepr v 2.7
Data analysis	EPR: mtsslSuite (ref. 64), DeerAnalysis 2018/21b(ref. 50), smFRET: all data analysis tools are established and described (Gouridis et al., NSMB 2015 & de Boer et al., eLife 2019 - ref 31/79; Gebhardt et al., bioRxiv 2020 - ref 68).

For manuscripts utilizing custom algorithms or software that are central to the research but not yet described in published literature, software must be made available to editors and reviewers. We strongly encourage code deposition in a community repository (e.g. GitHub). See the Nature Portfolio guidelines for submitting code & software for further information.

Data

Policy information about availability of data

All manuscripts must include a data availability statement. This statement should provide the following information, where applicable:

- Accession codes, unique identifiers, or web links for publicly available datasets
- A description of any restrictions on data availability
- For clinical datasets or third party data, please ensure that the statement adheres to our policy

The data in this manuscript is provided as a Source Data File and datasets have been deposited in the Zenodo database. Accession codes are given in the Data availability section of the manuscript.

Field-specific reporting

Please select the one below that is the best fit for your research. If you are not sure, read the appropriate sections before making your selection.

☒ Life sciences ☐ Behavioural & social sciences ☐ Ecological, evolutionary & environmental sciences

For a reference copy of the document with all sections, see nature.com/documents/nr-reporting-summary-flat.pdf

Life sciences study design

All studies must disclose on these points even when the disclosure is negative.

Sample size	For FRET, n=3 independent experiments were conducted to be able to give an uncertainty for the calculated distances, as requested by the referee.<br>As is common and recommended by a recent expert guideline, multiple PELDOR/DEER distances on different double mutants of the same protein were were measured and interpreted as a whole to detect the influence of the ligand on the protein (Schiemann et al, 2021, JACS, https://doi.org/10.1021/jacs.1c07371). The measurements were further compared to structural models and the smFRET measurements. Samples that merited further investigations were repeatedly measured for example by titrating the ligand/cryoprotectant and by comparing the effect with measurements on a different double mutant. See also below in "Replication".
Data exclusions	All data was included in the analysis.
Replication	Multiple double mutants were measured for each example. Furthermore, the data were cross-validated by both methods. This is the central point of the paper. As requested by one of the referees, FRET data were measured in triplicate to provide error bars.<br>Several additional PELDOR/DEER experiments were conducted to verify the observed effect of cryoprotectant on the MalE distance distribution: We measured one sample with half the amount of d-Etgly cryoprotectant (25%), one sample with 25% d-Glycerol and one sample without cryoprotectant. In addition, we replicated the effect by using a different mutant from our library (MalE36/352), which was also measured without cryoprotectant.<br>Further, two MalE double mutants (29/352, 36/352) were measured at increased ligand concentration (10 mM) to check if this influences the observed effect of incomplete closure. The observation was consistent throughout all these measurements.<br>For SBD2 we performed several control experiments by increasing the ligand concentration (10 mM) to check for full closure of the protein and decreased the protein concentration (1.5 µM instead of 15 µM) to investigate our observation of the presence of the closed conformation in the absence of ligand and of the small percentage of open conformation in the presence of ligand.<br>Our attempts at replication for both FRET and PELDOR/DEER were hence successful.
Randomization	This study developed over time during a long-standing collaboration of the two involved laboratories. It was hence not possible to implement randomization.
Blinding	This study developed over time during a long-standing collaboration of the two involved laboratories. It was hence not possible to implement blinding

Reporting for specific materials, systems and methods

We require information from authors about some types of materials, experimental systems and methods used in many studies. Here, indicate whether each material, system or method listed is relevant to your study. If you are not sure if a list item applies to your research, read the appropriate section before selecting a response.

Materials & experimental systems

n/a	Involved in the study	
☒	☐	Antibodies
☒	☐	Eukaryotic cell lines
☒	☐	Palaeontology and archaeology
☒	☐	Animals and other organisms
☒	☐	Human research participants
☒	☐	Clinical data
☒	☐	Dual use research of concern

Methods

n/a	Involved in the study	
☒	☐	ChIP-seq
☒	☐	Flow cytometry
☒	☐	MRI-based neuroimaging
